# Multicenter prospective in vivo study of an endocytoscope system (ECS) for superficial esophageal cancer

**DOI:** 10.1007/s00535-021-01810-2

**Published:** 2021-07-25

**Authors:** Shuko Morita, Kenichi Goda, Tomonori Yano, Mitsuru Kaise, Mototsugu Kato, Haruhiro Inoue, Yasumasa Niwa, Shinya Kodashima, Ryoji Miyahara, Atsushi Ochiai, Masahiro Ikegami, Shigeharu Hamatani, Tadakazu Shimoda, Yasuo Ohkura, Junko Aida, Yukihiro Nakanishi, Kenichi Yoshimura, Hideki Ishikawa, Kaiyo Takubo, Manabu Muto

**Affiliations:** 1 Department of Gastroenterology, Kobe City Hospital Organization Kobe City Medical Center General Hospital, 2-1-1 Minatojima-Minamimachi, Chuo-ku, Kobe, Hyogo 650-0047 Japan; 2 Department of Gastroenterology, Dokkyo Medical University, 880 Kitakobayashi, Mibu, Shimotsugagun, Tochigi 321-0293 Japan; 3 Department of Gastroenterology and Endoscopy, National Cancer Center Hospital East, 6-5-1 Kashiwanoha, Kashiwa-shi, Chiba 277-8577 Japan; 4 Department of Gastroenterology, Nippon Medical School, Graduate School of Medicine, 1-1-5 Sendagi, Bunkyo-ku, Tokyo 113-8602 Japan; 5 National Hospital Organization Hakodate National Hospital, 18-16 Kawaharacho, Hakodate city, Hokkaido 041-8512 Japan; 6 Digestive Diseases Center, Showa University Koto-Toyosu Hospital, 5-1-38 Toyosu, Koto-ku, Tokyo 135-8577 Japan; 7 Department of Endoscopy, Aichi Cancer Center Hospital, 1-1 Kanokoden, Chikusa-ku, Nagoya 464-8681 Japan; 8 Department of Medicine, Teikyo University School of Medicine, 2-11-1 Kaga, Itabashi-Ku, Tokyo 173-8606 Japan; 9 International Medical Center, Fujita Health University Hospital, 1-98 Dengakugakubo, Kutsukake-cho, Toyoake, Aichi 470-1192 Japan; 10 National Cancer Center, Exploratory Oncology Research & Clinical Trial Center, 6-5-1 Kashiwanoha, Kashiwa-shi, Chiba-ken 277-8577 Japan; 11 Department of Pathology, The Jikei University School of Medicine, 3-19-18 Nishi-Shimbashi, Minato-ku, Tokyo 105-8471 Japan; 12 Department of Pathology, Shizuoka Cancer Center, 1007 Shimonagakubo, Nagaizumi-cho, Sunto-gun, Shizuoka Prefecture 411-8777 Japan; 13 Pathology & Cytology Center, PCL JAPAN, 1361-1 Matoba, Kawagoe, Saitama 350-1101 Japan; 14 Research Team for Geriatric Pathology, Tokyo Metropolitan Institute of Gerontology, 35-2 Sakae-cho, Itabashi-ku, Tokyo 173-0015 Japan; 15 Department of Pathology, Moffitt Cancer Center, 12902 USF Magnolia Drive, Tampa, FL 33612 USA; 16 Hiroshima University Hospital, Hiroshima University, 1-2-3 Kasumi, Minami-ku, Hiroshima City, Hiroshima 734-8551 Japan; 17 Department of Molecular-Targeting Cancer Prevention, Graduate School of Medical Science, Kyoto Prefectural University of Medicine, Kyoto-shi, Kamigyo-ku Kajii-cho, Kawaramachi-Hirokoji, Kyoto, 602-8566 Japan; 18 Department of Therapeutic Oncology, Graduate School of Medicine, Kyoto University, 54 Kawahara-cho, Shogoin, Sakyo-ku, Kyoto, 606-8507 Japan

**Keywords:** Endocytoscope systems, ECS, Esophageal squamous cell carcinoma, Endoscopic histological diagnosis

## Abstract

**Background:**

Endocytoscope systems (ECS) can visualize cellular nuclei of the mucosa of the gastrointestinal tract and are predicted to provide real-time microscopic diagnosis. However, their practical diagnostic performance remains unclear. Therefore, we conducted a multicenter prospective study to evaluate the visualization of superficial esophageal neoplasm in vivo using an ECS, and its diagnostic capability.

**Methods:**

The study target was histologically confirmed squamous cell carcinoma (SCC) and high-grade intraepithelial neoplasia (HGIN). An integrated ECS was used to obtain ECS images. In each patient, three ECS images of cancerous and corresponding noncancerous regions were selected for evaluation. A pathological review board of five certified pathologists made the final diagnosis of the images. The primary endpoint was the sensitivity of ECS diagnosis by pathologists.

**Results:**

ECS images of 68 patients were assessed: 42 lesions were mucosal SCC, 13 were submucosal SCC, and 13 were HGIN. The rate of assessable images was 96% (95% CI 87.6–99.1). The sensitivity of ECS diagnosis by pathologists was 88% (95% CI 77.2–94.5).

**Conclusions:**

ECS can provide high-quality images of cancerous lesions and a high diagnostic accuracy by pathologists, and could be useful for real-time endoscopic histological diagnosis of SCC and HGIN.

**Trial registration:**

The UMIN Clinical Trials Registry Identification Number: 000004218

**Supplementary Information:**

The online version contains supplementary material available at 10.1007/s00535-021-01810-2.

## Introduction

Virtual biopsy is an ideal approach for making rapid histological diagnoses, as it can shorten the time for diagnosis, reduce the risk of bleeding or fibrotic changes after biopsy, and reduce the amount of time and work of pathologists.

An endocytoscope system (ECS) has a magnifying power of 1100-fold. Using ECS, Kumagai et al [1] and Inoue et al [2] succeeded in visualizing the cell nuclei of the mucosa of the gastrointestinal tract. Furthermore, Inoue et al [3] reported that endocytoscopic images were useful for diagnosis of esophageal squamous cell neoplasia. Fujishiro et al [4] demonstrated the similarities between ex vivo endocytoscopic images and horizontal histological images of cancerous and normal squamous cells in the esophagus. However, the in vivo diagnostic ability of ECS has not been evaluated in a prospective study. In addition, it is important to determine whether pathologists can make an accurate diagnosis of cancer using ECS images.

On the basis of these key issues, we addressed two clinically important questions in a multicenter prospective study. First, can endoscopists obtain high quality ECS images during routine endoscopic procedures? Second, can pathologists diagnose malignant cells using ECS images, without relying on hematoxylin and eosin (H&E) staining of biopsy specimens?

## Methods

### Subjects

This study examined superficial esophageal lesions. Inclusion criteria were as follows: (1) histologically confirmed squamous cell carcinoma (SCC) or high-grade intraepithelial neoplasia (HGIN) by biopsy specimen; (2) the suspected depth of the lesion was limited to within the mucosa or shallow submucosal layer (SM1); (3) the lesion was larger than 10 mm in diameter; and (4) subjects were patients aged 20 years or older. We excluded patients who had been treated with radiation therapy or chemotherapy, because such treatments may have affected the findings. Other exclusions were (1) the lesions located in the cervical esophagus because of difficulty in holding the scope and potential serious complications (e.g., aspiration) interfere during the examination, (2) the use of thrombolytic agents because it interfere taking biopsy specimen.

### Equipment

**Fig. 1:**
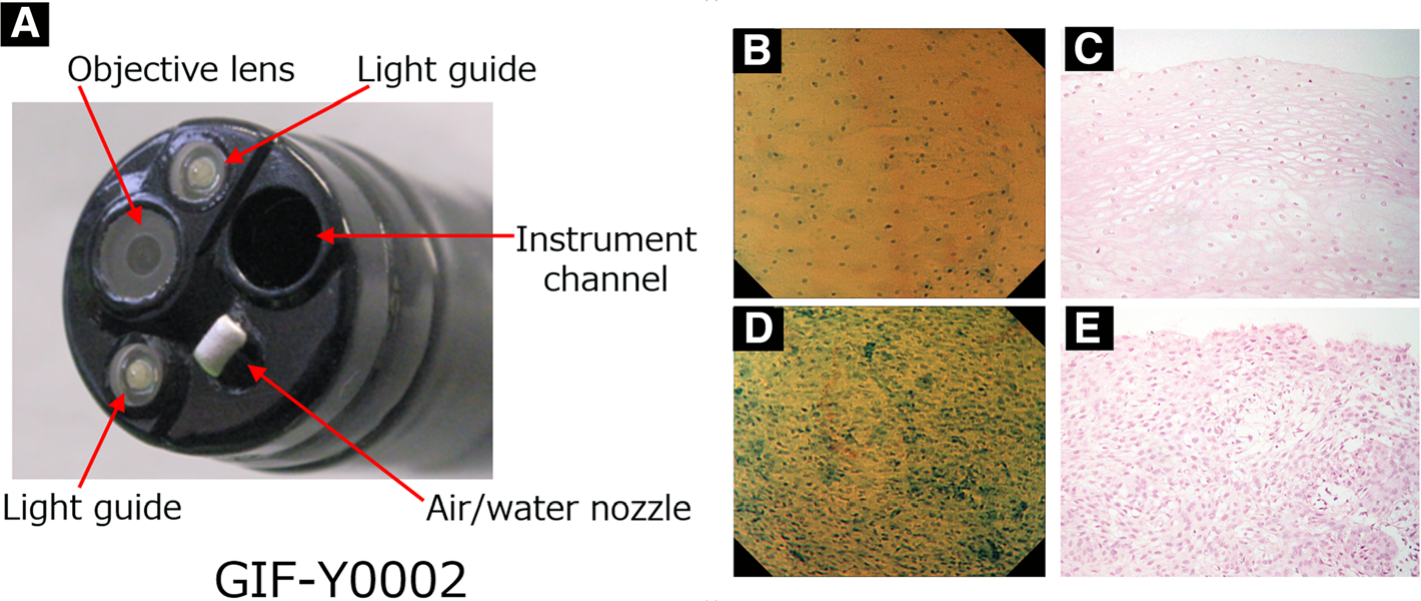
Endocytoscopic system (ECS) and images. **A** Endocytoscopic system (ECS). **B** Endocytoscopic image (1% methylene blue. × 380 on a 19-inch monitor) of non-cancer lesion. **C** Histologic image (H and E stain) of non-cancer lesion. **D** Endocytoscopic image of cancer lesion. **E** Histologic image (H and E stain) of cancer lesion

The prototype endocytoscope used was 13.6 mm in diameter. It consisted of one lens, two light guides, and a 3.2 mm working channel (Olympus Medical Systems, Tokyo, Japan) (Fig. 1). A video processor (CV-260, CV-260SL; Olympus Medical Systems) and a light source (CLV260NBI, CLV-260SL; Olympus Medical Systems) were used in this study.

This prototype endocytoscope can consecutively increase the image magnification up to 380-fold (on a 19-inch monitor) with a hand lever, which covers a 700 × 600 μm area of tissue and the light focusing depth ranges from 0 to 50 μm. Using digital 1.6-fold magnification, the magnifying capability is up to 600-fold magnification (on a 19-inch monitor), covering a 440 × 380 μm area.

### ECS images

Before the ECS images were obtained, the target lesion was soaked for 60 s in 1% methylene blue solution. After washing with water, the lesions were observed and images collected by ECS.

### Study design

This multi-center prospective study was conducted at nine centers in Japan and the study flow is summarized in supplementary Fig. 1. The primary endpoint assessed was the sensitivity of the procedure, namely the proportion of the ECS images that were assessable, high-quality images and could be diagnosed as esophageal cancer by five certified pathologists. The secondary endpoints were as follows: (1) the rate of assessable high-quality images in the total number of examined images; (2) interobserver agreement between the five pathologists; (3) the specificity of ECS diagnosis by the five pathologists; and (4) interobserver agreement between pathologists and endoscopists.

Before beginning of this study, 15 participating endoscopists observed the superficial esophageal SCC, which was eligible lesion for this study, by ECS and selected the demonstrable ECS images of cancerous or noncancerous lesions for central review. During the examination, six ECS images were obtained from each case (three images of cancerous and corresponding noncancerous regions, respectively). These images were used for endoscopists’ diagnosis to compare the difference to the pathologists. These ECS images were stored for central evaluation of image quality and suitability for diagnosis by pathologists. Biopsy specimens from cancerous and noncancerous regions were taken from target lesions. If the lesion was small, a biopsy was not taken to avoid histological change. Biopsy or endoscopically resected specimens were used for the histological evaluation. Specimens were stained with H&E. Histologic evaluation was performed by central review by three independent, certified pathologists (K.T., J.A., Y.N.) to make gold-standard diagnoses of the specimens. The pathological diagnostic criteria were based on the Vienna classification of gastrointestinal epithelial neoplasia. [5] Noninvasive, high-grade neoplasia and invasive neoplasia were diagnosed as carcinoma; negative for neoplasia/dysplasia, indefinite for neoplasia/dysplasia, and noninvasive low-grade neoplasia were diagnosed as noncarcinoma.

Five certified pathologists (M.I., Y.O., A.O., T.S., S.H.) independently evaluated the six images of each case and diagnosed them using a web review system. These pathologists did not participate in central review to make gold-standard pathological diagnosis. Before the beginning of the study, all participating pathologists were trained using ECS images other than registered cases. They were blinded to the clinical or histological information. Each pathologist independently accessed the Medical Stream website via a personal computer, and independently evaluated the ECS images within a 1-week period (Supplementary Fig. 2). ECS images were classified into five grades based on the criteria reported by Inoue et al [2] (endocytoscopic atypia (ECA) I–V). In this study, ECA I–III were interpreted as noncarcinoma, and ECA IV or V as carcinoma (Fig. 1).

Initially, the five pathologists evaluated the quality of the ECS images and thereafter classified them. If four or five pathologists evaluated the image as “assessable with high quality,” it was assigned to the group of assessable images with high quality. If four or five pathologists diagnosed the ECS image as “cancer,” it was defined as a cancerous lesion. If consensus was not reached regarding evaluation and classification, a final decision was made after discussion between the five pathologists.

The protocol of this study was approved by the Institutional Review Board of Kyoto University and each participating center. Written informed consent was obtained from all participants. The UMIN Clinical Trials Registry Identification Number for this study is 000004218.

### Statistical analysis

Statistical analysis was performed by a third party. Sample size was determined by setting the threshold and expected sensitivity for the primary endpoint at 75% and 90%, respectively. These settings predicted a sample size of 65 cases; to allow for some ineligible cases, the sample size was set at 70 cases.

The sensitivity, specificity, and the rate of assessable images are presented as percentages with 95% confidence intervals (CIs). Interobserver agreement (between the five pathologists, and between pathologists and endoscopists) was calculated using κ statistics for the following variables: (1) the evaluation of ECS images among the five pathologists; and (2) the evaluation of ECS images between pathologists and endoscopists. Interpretation of the κ values was performed according to Landis and Koch. [6] A κ value < 0 was considered as no agreement, 0–0.20 as slight, 0.21–0.40 as fair, 0.41–0.60 as moderate, 0.61–0.80 as substantial, and 0.81–1.00 as almost perfect agreement. All probability values calculated were two-sided, and *p* < 0.001 was considered significant.

## Results

**Fig. 2:**
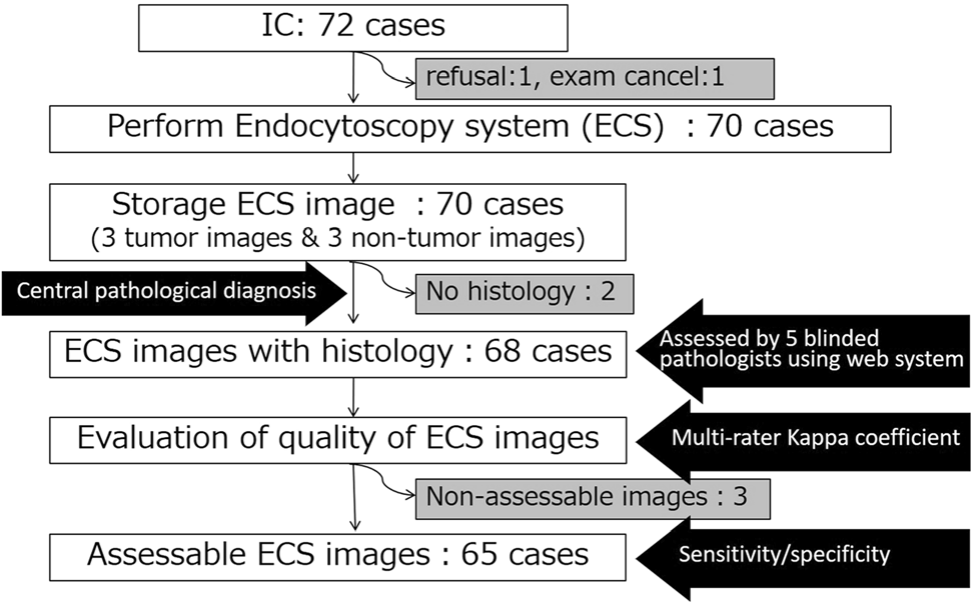
Flow chart for evaluation of superficial esophageal squamous cell carcinoma

A total of 72 patients with SCC or HGIN were enrolled in this study between May 2011 and January 2012. Four patients were excluded for the following reasons: one patient refused to participate, one canceled the ECS examination, and two did not have a biopsy specimen collected (Fig. 2). The remaining 68 patients (59 men and 9 women) with a median age of 67.5 (range, 45–85 years) were analyzed. The median tumor size was 22.5 mm (range, 10–60 mm); 42 lesions were mucosal SCC, 13 lesions were submucosal SCC, and 13 were HGIN (Table 1).

**Table 1:** Patient characteristics

	(*N* = 68)
Median age (range) years	67.5 (45–85)
Gender M: F	59: 9
Location	
Upper	3
Mid	40
Lower	25
Median size (range) mm	22.5 (10–60)
Histology	
SCC	55
HGIN	13

The sensitivity of the ECS diagnosis was 88% (57/65) (95% CI 77.2–94.5). Three cases were evaluated as low-quality images by the pathologists. Thus, the rate of assessable images with high quality in the total number of examined images was 96% (65/68) (95% CI 87.6–99.1) (Table 2). The specificity of the ECS diagnosis was 100% (95% CI 94.5–100) (Table 3). The multi-rater κ coefficient between the five blinded pathologists was 0.76 (*p* < 0.001) (Table 4). The multi-rater κ coefficient between pathologists and endoscopists was 0.88 (95% CI 0.80–0.96) (Table 5).

**Table 2:** Rate of assessable ECS images (*N* = 68)

ECS images	*N*	Rate	95% CI
Assessable	65	96%	87.6–99.1
Non-assessable	3

**Table 3:** Sensitivity and specificity of ECS diagnosis

	*N*	Rate (%)	95% CI
Sensitivity	65	88	77.2–94.5
Specificity	65	100	94.5–100

**Table 4:** Multi-rater *κ* coefficient between five blinded pathologists

	Pathologist					
		1	2	3	4	5
Pathologist	1		0.86	0.69	0.78	0.78
	2			0.69	0.82	0.83
	3				0.68	0.65
	4					0.85
	5					

Multi-rater kappa coefficient 0.76, *p* < 0.001. Landis–Koch criteria were: < 0 as no agreement; 0–0.20 as slight; 0.21–0.40 as fair; 0.41–0.60 as moderate; 0.61–0.80 as substantial; and 0.81–1.00 as almost perfect agreement

**Table 5:** Multi-rater *κ* coefficient between pathologists and endoscopists (*N* = 65)

Endoscopists	Pathologists		
	Cancer	Non-cancer	Total
Cancer	57	8	65
Non-cancer	0	65	65

Kappa coefficient = 0.88 (95% CI 0.80–0.96)

## Discussion

In this multi-center prospective study, we demonstrated for the first time that high-quality ECS images could be obtained in 96% during the endoscopic procedures, and that the sensitivity of the cancer diagnosis by ECS in certified pathologists was 88%. These results indicated that the pathologists could diagnose SCC using ECS images. The multi-rater κ coefficient between the five blinded pathologists was 0.76, and the multi-rater κ coefficient between pathologists and endoscopists was 0.88. These results have clinical importance as precise diagnosis by in vivo ECS imaging and also provide the possibility for real-time, on-site diagnosis (virtual biopsy) by both endoscopists and pathologists.

To get a clear ECS image, it is necessary to hold the endoscope firmly still. Although esophageal peristalsis, cardiac pulsation, and respiration movement may interfere with the procurement of high-quality ECS images in vivo, the assessable rate of ECS images in this study was very high (96%), meaning that in vivo ECS imaging can be applied in clinical practice.

While IEE including NBI is now recognized to be useful technique for diagnosing esophageal cancer and it is widely accepted [7], pathological conformation is still required in clinical practice. Furthermore, when treatment is indicated, histological confirmation is essential. However, biopsies cause fibrosis and make endoscopic treatment difficult. And, it is time consuming because conventional pathological diagnosis need to prepare H&E stained tissue sections. So if ECS diagnosis would be comparable to pathology as vertical biopsy, it is beneficial to decide on a treatment plan quickly without causing fibrosis.

Real-time, on-site diagnosis is clinically important for deciding the treatment of SCC. Especially, elderly patients have a high risk of bleeding after biopsy due to many complications and/or the consumption of prescribed anticoagulants for cerebro-cardiovascular disease. Even for the general population, there is a risk of bleeding by biopsy. Notably, in vivo ECS diagnosis may make it possible to avoid unnecessary and excessive biopsy procedures.

Kumagai Y et al. previously investigated whether the pathologist could evaluate nuclear density and nuclear abnormality in the ECS images and also whether biopsy histology can be omitted [8]. They reported that pathologist agreed to omit biopsy histology in 84% of cases which observed by 1125-fold magnification ECS. However, it decreased to 66% of cases which observed by 450-fold magnification ECS. While direct comparison between Kumagai’s report and our results was difficult, our data of high sensitivity (88%) by pathologists to diagnose cancer was comparable while ECS used in this study was 600-fold magnification. The 1125-fold magnification ECS was a prove type and had to pass through the active channel of the main endoscope. In contrast, ECS system used in this study was integrated with conventional video endoscope. Then, it was easy to change ECS observation. This had a great merit for the endoscopist, because prove type was complicated to manipulate and time consuming. Then, our results are supportive of clinical implementation of ECS.

This study has several limitations. First, this study targeted lesions that were already diagnosed as SCC or HGIN. As a first step for evaluation of ECS images for SCC diagnosis, we selected these lesions because the pathological diagnosis of low grade dysplastic lesions has been difficult for pathologists. As a result, we demonstrated that pathologists can distinguish a cancerous lesion from a noncancerous lesion using ECS images. As a next step, we can attempt to differentiate undiagnosed lesions with in vivo real-time ECS imaging. Second, as the ECS can visualize the vertical area within a depth of 50 μm from the surface, we cannot diagnose invasive cancer deeper than 50 μm. Realistically, methylene blue can stain cells that exist at the mucosal surface only and don't penetrate to 50 µm. Then, we can only observe the cells at the tumor surface. This disadvantage should be overcome by providing supportive, routine white light imaging or Narrow Band Imaging or endoscopic ultrasound. Third, the ECS used in this study become old. The latest ECS is now commercially available. This fourth-generation ECS can continuously increase the optical magnification up to 500-fold and after 1.8-time digital magnification up to 900-fold. In addition, it succeeded in achieving high-vision, which enables us to capture the nuclear morphology more clearly. The outer diameter has been further reduced to 9.7 mm. Using this latest ECS, we expected further high performance of the diagnosis of ECS in clinical practice.

In conclusion, in vivo ECS can provide images of high quality that are sufficient for pathological diagnosis and that produce a high diagnostic yield of cancerous lesions by pathologists. In vivo ECS imaging could be useful for real-time endoscopic histological diagnosis, vertical biopsy, of superficial esophageal SCC and HGIN.

## Supplementary Information

Below is the link to the electronic supplementary material.

Supplementary Fig. 1. Flowchart for evaluation of superficial esophageal squamous cell carcinoma (TIF 987 KB)

Supplementary Fig. 2. The schedule of ECS-image evaluation by pathologists (TIF 381 KB)

## References

[1] Kumagai Y, Monma K, Kawada K (2004). Magnifying chromoendoscopy of the esophagus: in-vivo pathological diagnosis using an endocytoscopy system. Endoscopy.

[2] Inoue H, Kazawa T, Satodate H (2004). In vivo observation of living cancer cells in the esophagus, stomach, and colon using catheter-type contact endoscope, “Endo-Cytoscopy system”. Gastrointest Endosc Clin N Am.

[3] Inoue H, Sasajima K, Kaga M (2006). Endoscopic in vivo evaluation of tissue atypia in the esophagus using a newly designed integrated endocytoscope: a pilot trial. Endoscopy.

[4] Fujishiro M, Takubo K, Muto M (2007). Potential and present limitation of endocytoscopy in the diagnosis of esophageal squamous-cell carcinoma: a multicenter ex vivo pilot study. Gastrointest Endosc.

[5] Schlemper RJ, Riddell RH, Kato Y (2000). The Vienna classification of gastrointestinal epithelial neoplasia. Gut.

[6] Landis JR, Koch GG (1977). The measurement of observer agreement for categorical data. Biometrics.

[7] Muto M, Minashi K, Yano T (2010). Early Detection of Superficial Squamous Cell Carcinoma in the Head and Neck Region and Esophagus by Narrow Band Imaging: A Multicenter Randomized Controlled Trial. Journal of Clinical Oncology.

[8] Kumagai Y, Kawada K, Yamazaki S (2009). Endocytoscopic observation for esophageal squamous cell carcinoma: can biopsy histology be omitted?. Dis Esophagus.

