# Supplementary figures and images for: Multicenter prospective in vivo study of an endocytoscope system (ECS) for superficial esophageal cancer
Source: J Gastroenterol. 2021 Jul 25;56(9):808–13. doi: 10.1007/s00535-021-01810-2 (PMC8370913; doi:10.1007/s00535-021-01810-2)

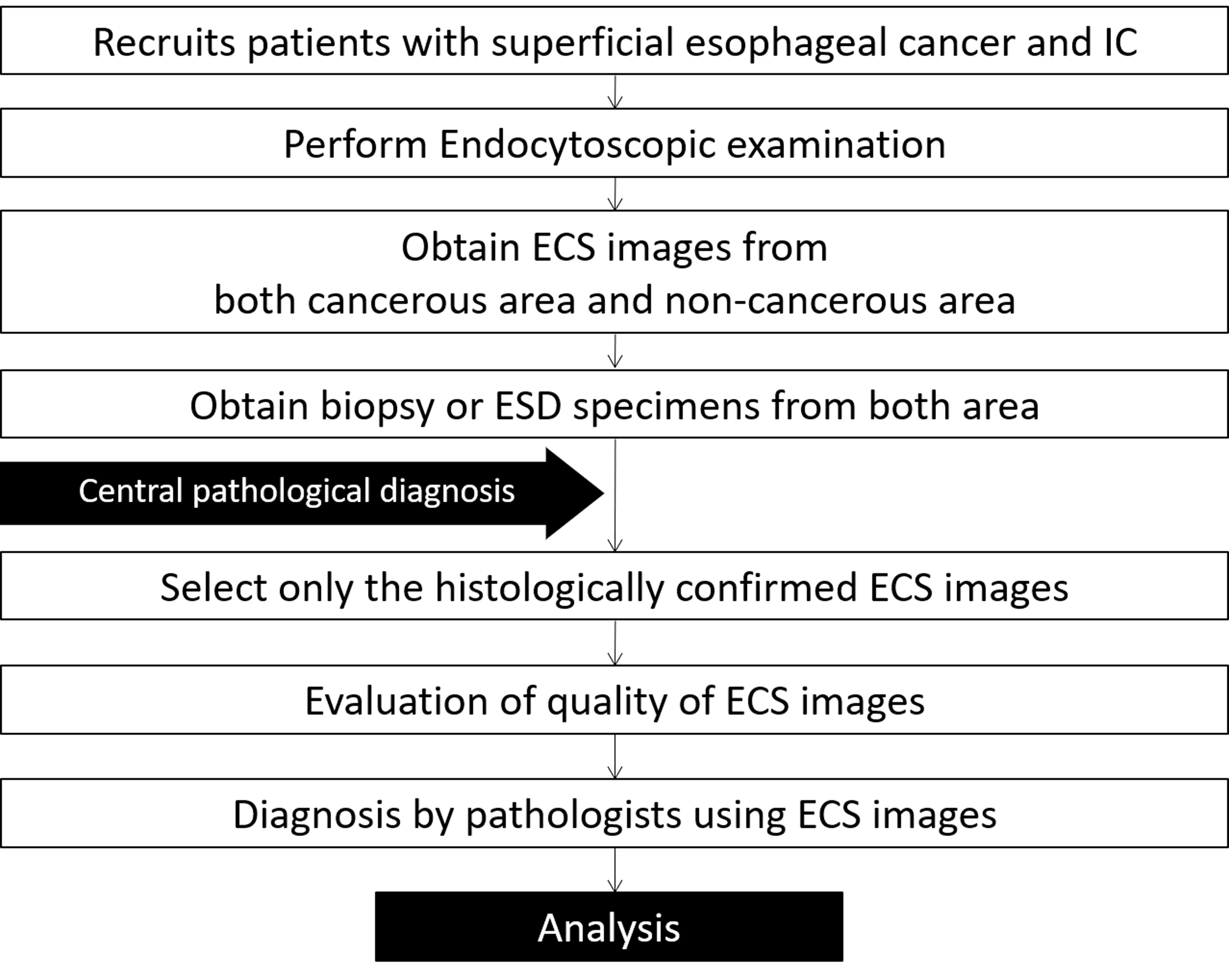

Supplement: Supplementary file 1 — Supplementary Fig. 1. Flowchart for evaluation of superficial esophageal squamous cell carcinoma (TIF 987 KB) [file 535_2021_1810_MOESM1_ESM.tif]

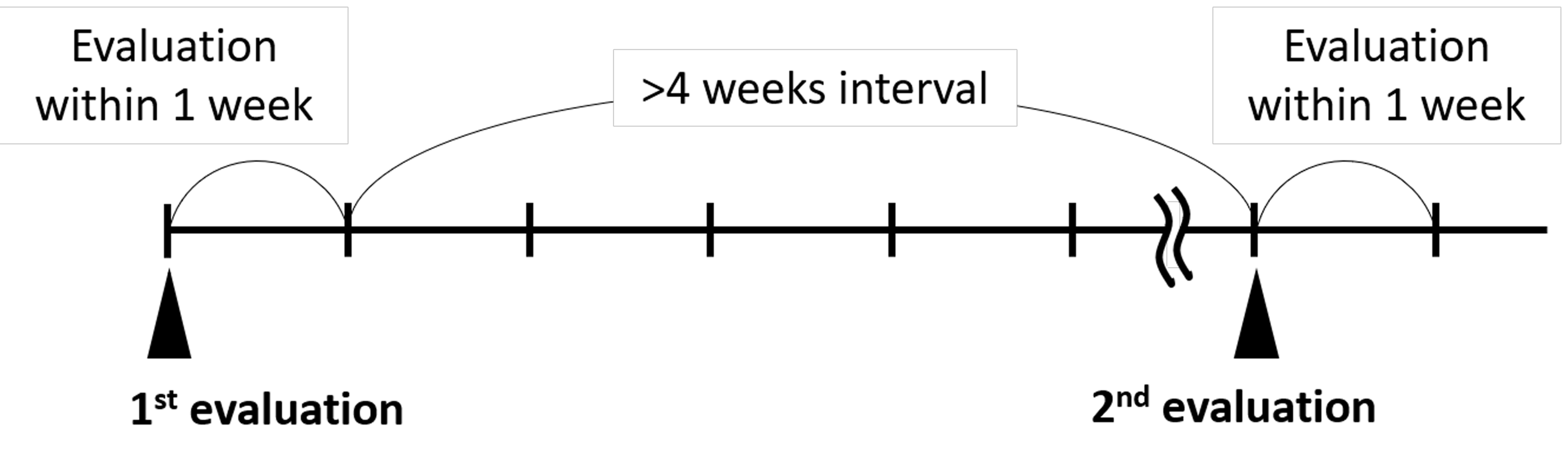

Supplement: Supplementary file 2 — Supplementary Fig. 2. The schedule of ECS-image evaluation by pathologists (TIF 381 KB) [file 535_2021_1810_MOESM2_ESM.tif]
